# Infant mortality and ethnicity in an indigenous European population: Novel evidence from the Finnish population register

**DOI:** 10.1038/srep04214

**Published:** 2014-02-27

**Authors:** Jan Saarela, Fjalar Finnäs

**Affiliations:** 1 University of Helsinki and Åbo Akademi University, Finland, Jan Saarela, Åbo Akademi University, PO Box 311, FIN-65101 Vasa, Finland; 2 Fjalar Finnäs, Åbo Akademi University, PO Box 311, FIN-65101 Vasa, Finland

## Abstract

We provide the first analyses of infant mortality rates by indigenous ethnic group in Finland, a country that has one of the lowest relative numbers of infant deaths in the world. Using files from the Finnish population register, we identified both of the parents of children born in the period from 1975–2003 according to ethnic affiliation, socioeconomic profile, and demographic position. The infant mortality rate in homogamous Finnish unions is similar to that in homogamous Swedish unions, which reflects a lack of social disparities between the two groups. Surprisingly, infants from ethnically mixed unions have markedly lower mortality rates, with an adjusted rate ratio of 0.81 relative to homogamous Swedish unions (95% CI: 0.67–0.98). Although not empirically verified, we argue that the lower infant mortality rate in ethnically mixed unions may be due to lower levels of inbreeding, and hence related to historically low intermarriage rates between the two ethnic groups, remote consanguinities, and restricted inter-community gene flow.

Infant mortality is generally considered to reflect the overall health status of a population. A low rate is strongly associated with high levels of economic development, standards of living, social well-being, and environmental quality, as well as low rates of illness^1^. Since the mid-1970s, Finland, together with countries such as Sweden and Japan, has had the lowest infant mortality rate in the world. In 1976, the number of deaths among children aged less than 1 year per 1,000 live births was 9.3, compared with 9.4 in Japan and 8.3 in Sweden. In 2011, the corresponding rates were 2.3, 2.4, and 2.2, respectively^2^.

An overlooked aspect of infant mortality in Finland is whether there is any difference between the two indigenous ethnic groups, namely, Finnish speakers and Swedish speakers. The primary aim with the present study is to fill this gap in the literature. In contrast to many other countries, such as the United States, where infant mortality rates notably vary by mothers' ethnic origins^3^, there is no evident reason to expect any significant ethnic gradient in Finland. The two groups are equal with respect to living conditions and socioeconomic position, they live intermingled and are similar in physical appearance. Swedish speakers are also guaranteed the same constitutional rights as Finnish speakers, even though Swedish speakers constitute barely 6% of the total population.

Nevertheless, having achieved one of the world's lowest infant mortality rates does not confirm the nonexistence of an ethnic gradient. As in other countries with low infant mortality rates (see, e.g.,^4^,^5^), there is a social gradient. In the late 1980s, infants of Finnish mothers with a low socioeconomic position had nearly 40% higher death rates than infants of mothers with a high socioeconomic position^6^. Similar conclusions apply if maternal education is used as a proxy for social class^7^,^8^,^9^. Higher mortality rates have also been reported for infants of single mothers than for infants of mothers with a partner. However, for the offspring of mothers who live in a union, there is no difference in infant mortality according to marital status^10^. Social inequalities in infant health outcomes diminished further in the 1990s but still exist^11^, and variations in lifestyle markers are not the sole explanation for these inequalities. Smoking has been found to account for at most one-third of the differences in preterm births (births before the 37th gestational week) between socioeconomic groups^12^. Preterm births in turn constitute one of the most significant contributors to neonatal mortality (deaths within the first seven days of life), amounting to more than half of all infant deaths^13^.

From a methodological point of view, any thorough investigation of variation in infant mortality by ethnicity in Finland needs to account for both parents' ethnic affiliations. Children born into ethnically mixed unions are equally frequent as children with a homogamous Swedish background because approximately 40% of all Swedish speakers form a union with a Finnish speaker^14^. Before the 1950s, however, interethnic marriages were not common, as Swedish and Finnish speakers were geographically separated^15^.

The primary reason why there have been no previous studies of infant mortality by indigenous ethnic group in Finland is that the medical birth register does not include any variables that capture a mother's or father's ethnic affiliation^16^, and that information is not available from official vital statistics. In this study, we circumvent this problem by using files from the Finnish population register. These data are formed by combining information from Statistics Finland's longitudinal population census; longitudinal employment statistics; and registers of completed education and degrees, marriages and divorces, moves between dwellings, and births of children. Because information on the unique mother tongue of every individual in the Finnish population register is available, which serves as a marker of ethnic affiliation, we can classify each infant according to whether he or she has a homogamous Finnish, homogamous Swedish, or ethnically mixed background. This information is, together with parental socioeconomic and demographic variables, linked to records of all-cause infant deaths in the period from 1975–2003, meaning that we can provide the first estimates of infant mortality rates by indigenous ethnic group in Finland.

The control variables available are the mother's age at delivery, the calendar year of delivery, parity, plurality, the infant's sex, the area of residence, and each parent's educational level. The mother's age and the calendar year of delivery are classified into four equally sized categories. Plurality distinguishes single and multiple births. The area of residence consists of four broad regional categories. Educational level is separated into primary, lower secondary, upper secondary, or higher education. The distributions of the variables are reported in Table 1.

## Results

The unadjusted (raw) mortality rate of infants from homogamous Finnish unions for the entire period studied is nearly the same as that of infants from homogamous Swedish unions, or 4.9 per 1,000 live births, compared with 4.7 (Table 1). Infants from ethnically mixed unions, however, have markedly lower mortality rates than infants from homogamous unions (average of 3.9 per 1,000 live births). In relation to homogamous Swedish unions, this value corresponds to a ratio of 0.85.

Because the people in each homogamous ethnic group and those in mixed families are similar with respect to socioeconomic and demographic characteristics, adjusting for the variables in Table 1 has only a minor effect on the ethnic gradient (Table 2). The adjusted infant mortality rate in ethnically mixed unions relative to homogamous Swedish unions is 0.81 (95% CI: 0.67–0.98). In homogamous Finnish and homogamous Swedish unions, the adjusted rates are practically the same, and their rate ratio is 0.97 (95% CI: 0.85–1.09). More detailed analyses revealed that there is no significant difference in mortality between infants with a Swedish mother and a Finnish father and infants with a Finnish mother and a Swedish father.

The effects of the control variables are in line with previous studies (see^17^ for a review). The infant mortality rate of girls is 0.8 times that of boys, and the rate increases with maternal age at delivery, higher-order parity, and plurality and decreases over birth cohorts and with the maternal educational level. The same is true for the paternal educational level alone, but this variable has no effect after controlling for the mother's educational level. Differences across the broad geographic areas applied here are not statistically significant. Complementary analyses revealed that restricting the study population to families in Southern and Western Finland, where 95% of all Swedish speakers live, has no influence on the results.

## Discussion

By investigating infant mortality by indigenous ethnic group in Finland, we avoid several of the analytical pitfalls common in studies comparing natives with immigrants, such as the potential influence of migrant health selection and culture-related health behaviors typical of specific migrant groups^18^. Historically, Swedish and Finnish speakers have lived separately, but since the 1950s, a substantial proportion has intermarried. Currently, the number of children born with an ethnically mixed background is equal to the number born with a homogamous Swedish background. Homogamous Finnish, homogamous Swedish, and ethnically mixed families are of approximate sociodemographic status. Accordingly, we found no infant mortality difference between homogamous Swedish and homogamous Finnish unions.

Surprisingly, infants from ethnically mixed unions have almost 20% lower mortality rates than infants from ethnically homogamous unions, which is a notable difference. The effect size is equal to that of the difference in infant mortality between boys and girls^19^. If measured according to the currently modest infant mortality rate in Finland, the estimate corresponds to approximately 15 fewer deaths per annum in the total population. To put this figure into perspective, there were in the period 1993–2010 approximately 2,100 births per year in Finland diagnosed with a serious congenital anomaly. They accounted for 44% of all infant deaths, although an increasing number of pregnancies with congenital anomalies were selectively terminated^20^.

The quality of the available data is high. In spite of the relatively few infant deaths the estimates of the included socioeconomic and demographic variables are consistent. Small number problems might nevertheless explain some of the less expected findings, such as the slightly lower infant mortality rate of girls as compared to boys in the ethnically mixed group. Because the population register has no link to the medical birth register, we lack information regarding exposure before birth (such as maternal smoking), treatment before birth, delivery, and each newborn's short-term outcome. The causes of the attenuated infant mortality rate of ethnically mixed offspring can therefore only be hypothesized.

Differences in lifestyle factors and health behaviors related to environmental differences between the Swedish-speaking, Finnish-speaking, and ethnically mixed groups are an unlikely explanation. We found no difference in infant mortality rates between homogamous Swedish and homogamous Finnish unions, irrespective of whether we controlled for socioeconomic and demographic confounders. Furthermore, there exists no evidence which says that parents in ethnically mixed families have health behaviors that enhance their infants' survival as compared with mothers in homogamous Swedish or homogamous Finnish unions, such as a lower prevalence of maternal smoking. All available documentation rather points in the opposite direction. Social selection with respect to union and parenthood entry^21^ is consequently not likely. The separation risk of ethnically mixed unions of Swedish and Finnish speakers is higher than that of both homogamous Finnish and homogamous Swedish unions^14^, and infants in broken families generally fare much worse than other infants with respect to morbidity and mortality^4^,^10^. It is unreasonable to expect that people in a group with a high separation risk have social characteristics that enhance their infants' survival at an early stage.

Certain personal characteristics of parents must nevertheless influence the difference in infant mortality between homogamous and ethnically mixed unions. We think there are good reasons to argue that they may relate to genetic factors. In Finland, consanguineous unions (in clinical genetics, usually defined as unions between people who are biologically related as second cousins or closer) have not been a tradition or practiced by prescription. Remote consanguinities have been taken as evidence for the recessive inheritance of the Finnish diseases, however^22^. The Finnish disease heritage can be ascribed to the original patterns of human settlement, and genetic/demographic phenomena such as low local effective population sizes, founder effects, bottlenecks and genetic drift^23^. As in many other traditional societies, the population size has been small, and subgroups have been geographically isolated. Located at the edge of the inhabitable world, Finland is one of the best-studied genetic isolates. The small number of original founders followed by isolation, rapid expansion, and sampling of small immigrant groups from the main population, allowed the founder effect and genetic drift to mold the gene pool, especially in the regional sub-isolates^24^. Accordingly, we know that Swedish speakers in Finland differ genetically from Finnish speakers^21^,^22^,^23^, and there is ample evidence for substantial genetic diversity between the Western and Eastern subgroups^25^,^26^,^27^,^28^,^29^,^30^ that relates to susceptibility to specific diseases^31^,^32^,^33^,^34^,^35^,^36^,^37^. Hence even in the absence of preferential consanguinity, alleles that are rare in large populations can rapidly increase to a high frequency in a breeding pool of restricted size because of founder effects and random genetic drift^38^. In locations where population substructure exists due to ethnic or other divisions, the size of the breeding pool in each community is reduced. Marriage partner options thereby decrease, and the influence of genetic drift increases. The net result is random inbreeding, with rapid divergence of subgroup marker allele frequencies and specific mutations restricted to individual sub-communities or even to specific families^39^,^40^,^41^.

Because the intermarriage rate between Swedish and Finnish speakers has been historically low, the inter-community gene flow has been restricted. The coefficient of inbreeding (the proportion of homozygosity of all loci) of the present-day progeny of ethnically mixed unions must, by definition, therefore be lower than that of the offspring of homogamous unions. Although closer consanguineous marriages have been largely proscribed, multiple pathways of more distant consanguinity consequently seem inevitable given the size and structure of the population, which can result in a higher probability of recessive disorders being expressed. The depressed mortality rate of infants born of ethnically mixed unions, as observed here, cannot be considered as directly comparable to the excess death rate of infants from consanguineous unions, as reported elsewhere, which generally suggests an excess death rate of only a few percentage points^40^,^42^,^43^. We interpret our estimate as an ‘outbreeding’ effect, meaning that the value roughly measures the absence of inbreeding on infant mortality at the population level.

Unfortunately, using the available data, we cannot verify whether this argument is true. However, in low-mortality countries, the epidemiological transition has led to a situation in which genetic disorders account for increasing proportions of all morbidity and mortality. The less common a disorder is, the greater the influence of consanguinity is on the prevalence of the disorder^38^,^44^. Studies show that runs of homozygosity are more common in outbred individuals than was previously thought^45^, and there is also a general consensus that postnatal morbidity and mortality are elevated in the progeny of consanguineous unions^40^,^42^,^43^. In 1993–2010, only 3.5% of all births in Finland had congenital anomalies^20^. However, the mortality rate of infants with congenital anomalies was 43 per 1,000 live births, as compared to 2 per 1,000 live births for infants without congenital anomalies^20^,^46^,^47^. With an already low infant mortality rate, the proportional impact of genetic disorders on survival must consequently be high.

A notable limitation of our study is that we had no access to medical information. The characteristics of a birth or pregnancy, such as birth weight and gestational age, could be important mediating factors, although causality is not undisputed^48^. We also did not have access to any supporting data on stillbirths and the causes of infant deaths. Analyses of these issues would provide useful information on specific diseases^7^,^49^. It needs to be emphasized as well that there exists no empirical evidence of excess homozygosity in the Swedish and Finnish populations in Finland, and that the strict data protection practices at Statistics Finland, which is the institution that prepared the data, necessitates the use of samples and not data on the total population. The calculated reduction of almost 20 per cent in infant mortality of ethnically mixed unions is evidently subject to a considerable degree of uncertainty, since the small number of deaths leads to a relatively wide confidence interval around the point estimate. However, all data used come from random samples that are representative for the country's population. The offspring of ethnically mixed unions are growing in number in many societies world-wide. From the perspective of health and social equality, the lower mortality rate of infants with an ethnically mixed background in Finland is a novel finding that deserves further epidemiological scrutiny. We nevertheless acknowledge the partial nature of the data used, and the need for independent confirmation of the results in other equivalent populations.

## Methods

The data (permission TK-53-186-09) were derived from a set of population register files stored at Statistics Finland, known as ‘Palapeli’^50^. The files contain records from each quinquennial census in the period from 1970–2000 and from the year 2003. Reference individuals are linked to all of their co-residential partners and biological children. Information regarding births, deaths, marriages, union entries, separations, and migrations makes it possible to construct households and follow them over time. The census data include variables such as place of residence, household number, type of family, and position in the family. The infants under study were all born in the period from 1975–2003, constituting the offspring of people in a sample of 8% of all Finnish speakers born from 1920–1988, plus an identically constructed sample of 50% of Swedish speakers. Parents who do not appear as reference people can be identified through information regarding the timing of unions and data from the censuses. For 93.8% of all the infants, we found the second (non-reference) parent, whereas the residual group, with only one identified parent, was excluded from further analysis. To focus on standard families and births, infants born to women aged under 17 or 45 years or more, and to mothers with parity 7 or more were also excluded.

The observations were weighted according to the sampling proportion of the reference individuals. These weights were additionally adjusted for multiple occurrences of infants, which occurs if both parents appear as reference individuals. The total number of unweighted, unique observations was 312,796, and the number of infant deaths was 1,513.

Infant mortality rates were estimated using the complex-samples Cox regression module in SPSS (Statistical Package for the Social Sciences) 19.0, adjusting for the effects of standard socioeconomic and demographic variables. Our focus was on the mortality rate ratio of infants born of homogamous Finnish unions, homogamous Swedish unions, and ethnically mixed unions.

## Figures and Tables

**Table 1:** Characteristics of the study population (variable distribution and distribution of deaths) and infant mortality rate by ethnic group

	Homogamous Finnish			Homogamous Swedish			Ethnically mixed		
	% of obser-vations	% of deaths	Infant mor-tality rate	% of obser-vations	% of deaths	Infant mor-tality rate	% of obser-vations	% of deaths	Infant mor-tality rate
Maternal age, years									
17–24	21.8	19.7	4.4	17.0	16.1	4.5	16.6	19.7	4.6
25–28	28.8	27.5	4.6	29.9	36.2	5.7	26.8	29.5	4.2
29–32	26.3	26.0	4.8	29.1	25.0	4.1	29.2	28.2	3.7
33–44	23.0	26.8	5.7	24.0	22.8	4.5	27.3	22.6	3.2
Parity									
1	40.6	37.1	4.5	38.5	31.3	3.8	41.4	28.2	2.6
2	36.0	35.2	4.8	36.8	37.9	4.9	36.4	47.1	5.0
3	16.1	17.0	5.1	17.5	19.2	5.2	15.9	17.3	4.2
4	5.1	6.9	6.6	5.2	6.7	6.2	4.8	5.3	4.3
5	1.6	2.2	6.7	1.5	3.6	11.6	1.2	2.1	7.1
6	0.7	1.6	11.1	0.7	1.3	9.7	0.4	0.0	0.0
Plurality									
1	97.4	88.1	4.4	97.4	82.6	4.0	97.3	87.5	3.5
2+	2.6	11.9	22.5	2.6	17.4	31.7	2.7	12.5	17.8
Infant's sex									
Boy	51.0	57.7	5.5	51.6	52.2	4.8	51.6	49.2	3.7
Girl	49.0	42.3	4.2	48.4	47.8	4.7	48.4	50.8	4.0
Year of delivery									
1975–1981	25.4	33.7	6.5	25.7	35.3	6.5	24.2	35.4	5.6
1982–1988	25.6	27.4	5.2	24.6	22.3	4.3	24.8	27.1	4.2
1989–1995	25.1	23.7	4.6	25.5	24.6	4.6	26.2	25.3	3.7
1996–2003	23.9	15.2	3.1	24.2	17.9	3.5	24.7	12.2	1.9
Maternal education									
Primary	17.4	21.9	6.1	16.1	18.8	5.5	19.1	30.9	6.2
Lower secondary	44.7	43.8	4.8	39.6	41.1	4.9	36.0	35.4	3.8
Upper secondary or higher	37.9	34.3	4.4	44.3	40.2	4.3	44.9	33.8	2.9
Paternal education									
Primary	23.2	26.3	5.5	25.1	24.8	4.7	25.7	25.5	3.8
Lower secondary	44.8	43.7	4.8	36.3	33.9	4.4	35.5	41.0	4.4
Upper secondary or higher	32.0	30.0	4.6	38.5	41.3	5.1	38.8	33.5	3.3
Area of residence in Finland									
Southern or Western	39.6	36.7	4.5	99.5	99.6	4.7	92.6	95.2	4.0
Central	26.1	27.8	5.2	0.2	0.4	9.7	3.5	3.2	3.5
Eastern	18.9	20.2	5.2	0.2	0.0	0.0	2.8	0.5	0.7
Northern	15.4	15.3	4.8	0.0	0.0	0.0	1.1	1.1	3.6
Total	100.0	100.0	4.9	100.0	100.0	4.7	100.0	100.0	3.9
Weighted number of infants	2,788,442			94,592			97,578		
Unweighted number of infants	234,761			47,296			30,739		
Unweighted number of infant deaths	1,141			224			148		

**Table 2:** Rate ratios of infant mortality with 95% confidence intervals

	RR	(95% CI)
Ethnicity		
Homogamous Finnish	0.97	(0.85–1.09)
Homogamous Swedish	1	
Mixed	0.81	(0.67–0.98)
Maternal age, years		
17–24	1	
25–28	1.12	(0.96–1.30)
29–32	1.15	(0.98–1.35)
33–44	1.33	(1.12–1.57)
Parity		
1	1	
2	0.99	(0.88–1.12)
3	0.98	(0.84–1.15)
4	1.17	(0.94–1.46)
5	1.18	(0.83–1.69)
6	2.03	(1.34–3.08)
Plurality		
1	1	
2+	5.25	(4.50–6.14)
Infant's sex		
Boy	1	
Girl	0.77	(0.70–0.86)
Year of delivery		
1975–1981	1	
1982–1988	0.79	(0.69–0.90)
1989–1995	0.69	(0.60–0.79)
1996–2003	0.46	(0.39–0.54)
Maternal education		
Primary	1	
Lower secondary	0.86	(0.75–0.98)
Upper secondary or higher	0.82	(0.71–0.96)
Paternal education		
Primary	1	
Lower secondary	1.01	(0.89–1.15)
Upper secondary or higher	0.97	(0.83–1.12)
Area of residence in Finland		
Southern or Western	1	
Central	1.14	(1.00–1.29)
Eastern	1.11	(0.96–1.28)
Northern	1.05	(0.90–1.23)

All estimates are from the same model.
